# Complete Genome Sequence of *Enterococcus faecium* Commensal Isolate E1002

**DOI:** 10.1128/genomeA.00113-16

**Published:** 2016-03-17

**Authors:** Hanne L. P. Tytgat, François P. Douillard, Pia K. Laine, Lars Paulin, Rob J. L. Willems, Willem M. de Vos

**Affiliations:** a Wageningen University, Laboratory of Microbiology, Wageningen, The Netherlands; b University of Helsinki, Department of Veterinary Biosciences, Helsinki, Finland; c University of Helsinki, Institute of Biotechnology, Helsinki, Finland; d University Medical Center Utrecht, Medical Microbiology, Utrecht, The Netherlands; e RPU Immunobiology, Department of Bacteriology and Immunology, Haartman Institute, University of Helsinki, Helsinki, Finland

## Abstract

The emergence of vancomycin-resistant enterococci (VRE) has been associated with an increase in multidrug-resistant nosocomial infections. Here, we report the 2.614-Mb genome sequence of the *Enterococcus faecium* commensal isolate E1002, which will be instrumental in further understanding the determinants of the commensal and pathogenic lifestyle of *E. faecium*.

## GENOME ANNOUNCEMENT

*Enterococcus faecalis* and *E. faecium* are considered as commensal bacteria of the human microbiota (1). However, some members of these species have evolved into important nosocomial threats, partially due to the acquisition of antibiotic resistance cassettes (2). In the past, *E. faecalis* was responsible for over 90% of all enterococcal infections, but since the 1980s the number of infections by *E. faecium* has increased and nowadays both species are equally frequently found as causative agents of nosocomial infections*.* Vancomycin-resistant enterococci (VRE), are now recognized as a global and increasing threat for human health (2).

Here, we report on the genome sequence of a commensal isolate of *E. faecium*, namely, E1002 (3, 4). This vancomycin-susceptible strain, with multilocus sequence type 54, was isolated from human feces of a nonhospitalized person in The Netherlands in 1998. Full genome sequencing of this bacterium and comparative genomic analysis including nosocomial strains will increase our insight into the determinants implicated in the commensal lifestyle of this species but will also increase our understanding of factors that play a role in pathogenesis and the acquisition of antibiotic-resistant cassettes.

*E. faecium* isolate E1002 was grown overnight anaerobically at 37°C in brain heart infusion (BHI) broth (Difco). Genomic DNA was extracted using the Wizard genomic DNA purification kit (Promega) as per manufacturer’s instructions. Genomic DNA sequencing of *E. faecium* isolate E1002 was then performed using Pacific Biosciences sequencing technology. Briefly, a library was constructed using the PacBio library kit and the size was targeted to 10 kb. Two single-molecule real-time (SMRT) cells were run using P6/C4 chemistry and 240 min video time. A total of 168 614 reads were assembled using the HGAP3 pipeline (Pacific Biosciences). A complete genome of 2 614 649 bp was obtained with a mean coverage of 478×.

Using Rapid Annotations using Subsystems Technology (RAST), 2,487 open reading frames (ORFs) were predicted in *E. faecium* E1002 (5). Interestingly, no plasmids were detected, in contrast with the fully sequenced hospital-strain *E. faecium* TX16 (6). Our results corroborate the report on the absence of the PilA-type pili in *E. faecium* E1002 (3), as the megaplasmid containing the PilA encoding pilin gene cluster-1 (PGC-1) is absent in this strain (7). The other three known PGCs were found present and were highly homologous to the *E. faecium* TX16 strain.

### Nucleotide sequence accession numbers.

The genome sequence project has been deposited in the European Nucleotide Archive (ENA) with the project number PRJEB12395 and the genome accession number is LN999844.
